# Supplementary figures and images for: N-mixture models provide informative crocodile (Crocodylus moreletii) abundance estimates in dynamic environments
Source: PeerJ. 2022 Mar 21;10:e12906. doi: 10.7717/peerj.12906 (PMC8944345; doi:10.7717/peerj.12906)

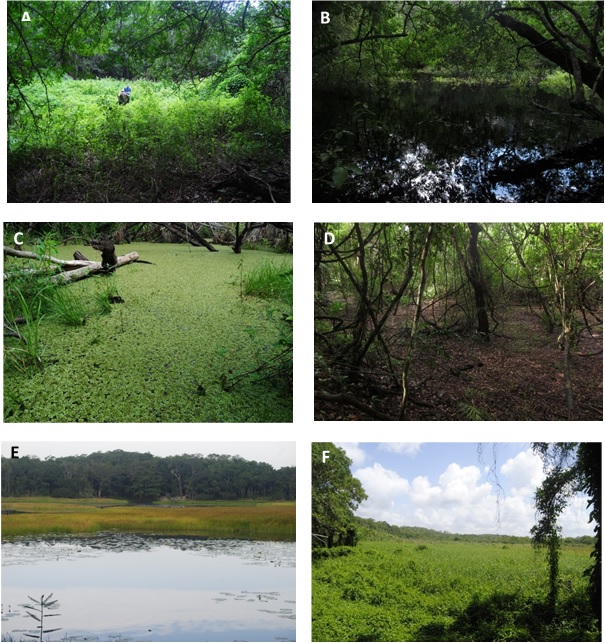

Supplement: Supplemental Information 1 — (A) Dry with High vegetation cover. (B) Same location with Full water level and moderate vegetation cover. (C) Drying with High vegetation. (D) Dry with Low vegetation. (E) Stable with Moderate vegetation. (F) Dry with High vegetation. For further details on waterbody general structure and photos regarding water level and vegetation please follow https://sites.google.com/view/baraonobrega-aguadas-calakmul/home (Barão-Nóbrega, 2019). [file peerj-10-12906-s001.jpg]

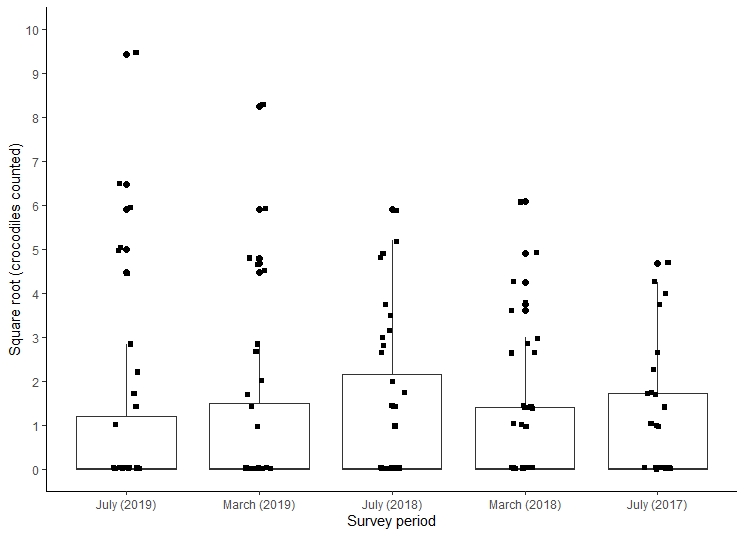

Supplement: Supplemental Information 2 [file peerj-10-12906-s002.jpeg]

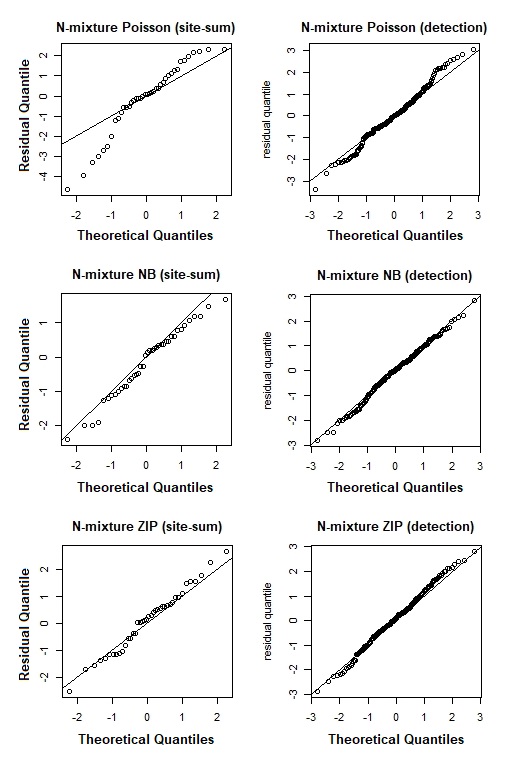

Supplement: Supplemental Information 3 — Under a good fit residuals should be close to the identity line. [file peerj-10-12906-s003.jpeg]

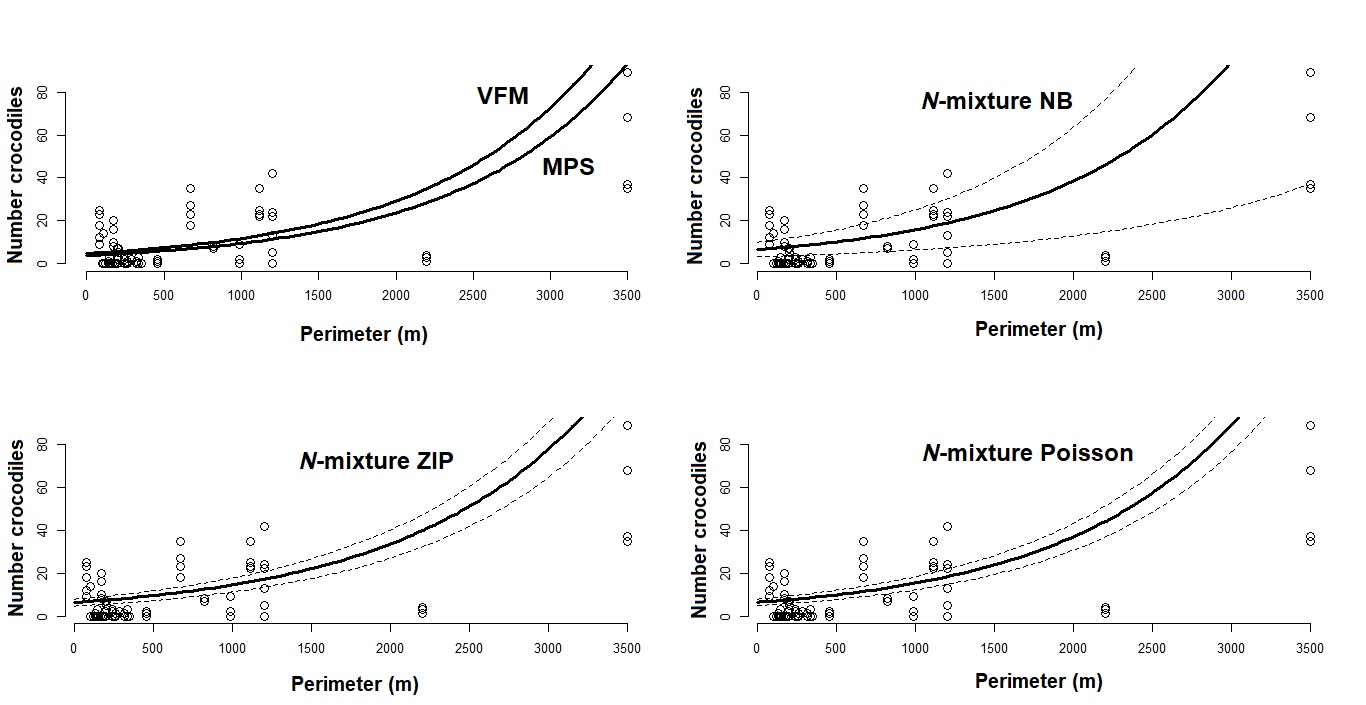

Supplement: Supplemental Information 4 — Black lines represent fitted values from estimations through King’s visible fraction method and Minimum population size using only baseline count data and binomial N-mixture modelling using a Poisson, a Negative Binomial (NB) and a Zero Inflated Poisson (ZIP) approach. Dashed lines in graphs represent the upper and lower confidence intervals. Open circles represent all our baseline crocodile count values (i.e. number crocodiles observed during the survey). [file peerj-10-12906-s004.jpeg]

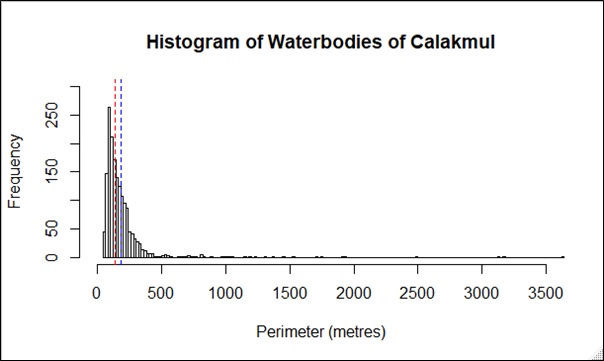

Supplement: Supplemental Information 5 — Dashed lines represent the median (red) and mean (blue) waterbody perimeter values. [file peerj-10-12906-s005.jpg]
